# Schistosomiasis among Recreational Users of Upper Nile River, Uganda, 2007

**DOI:** 10.3201/eid1605.091740

**Published:** 2010-05

**Authors:** Oliver W. Morgan, Gary Brunette, Bryan K. Kapella, Isabel McAuliffe, Edward Katongole-Mbidde, Wenkai Li, Nina Marano, Sam Okware, Sonja J. Olsen, W. Evan Secor, Jordan W. Tappero, Patricia P. Wilkins, Susan P. Montgomery

**Affiliations:** Centers for Disease Control and Prevention, Atlanta, Georgia, USA (O.W. Morgan, G. Brunette, B.K. Kapella, I. McAuliffe, W. Li, N. Marano, S.J. Olsen, W.E. Secor, J.W. Tappero, P.P. Wilkins, S.P. Montgomery); Ugandan Virus Research Institute, Entebbe, Uganda (E. Katongole-Mbidde); Ministry of Health, Kampala, Uganda (S. Okware)

**Keywords:** Schistosomiasis, vector-borne infections, travel, Uganda, Nile River, parasites, dispatch

## Abstract

After recreational exposure to river water in Uganda, 12 (17%) of 69 persons had evidence of schistosome infection. Eighteen percent self-medicated with praziquantel prophylaxis immediately after exposure, which was not appropriate. Travelers to schistosomiasis-endemic areas should consult a travel medicine physician.

Schistosomiasis, a parasitic infection caused by schistosome flukes, affects 207 million persons worldwide, mostly in sub-Saharan Africa (*1*). Schistosomiasis has been reported among travelers (*2*–*12*); 3 outbreaks have been reported among white-water rafters on the Omo River in Ethiopia (*2*,*7*,*10*). During September–November 2007, the Centers for Disease Control and Prevention (CDC) received reports of schistosome infection among travelers returning from white-water rafting on the Nile River, Jinja District, Uganda. Approximately 12,000 persons raft each year in Uganda, and local rafting companies believe that exposure to fast-moving white water during rafting and kayaking presents a low risk for schistosomiasis (C. McLeay, pers. comm.).

## The Study

During November 30–December 5, 2007, we enrolled a convenience sample of competitors and spectators attending the international Nile Freestyle Festival kayaking event and tourists on commercial rafting trips in Jinja District, Uganda. We administered a questionnaire to collect information about participants’ demographic characteristics, use of praziquantel (the antiparasitic drug treatment for schistosome infection), and exposure to fresh water. Three months after enrollment, we asked study participants who had had a negative or indeterminate result from a blood test for schistosome antibodies at the time of enrollment to complete an Internet-based questionnaire about freshwater exposures, health symptoms, and medical tests or treatments for schistosomiasis since enrollment.

We measured infection by collecting two 5-mL blood samples 3 months apart and testing them for evidence of schistosome antibody seroconversion. We tested for presence of schistosome-specific antibodies using an ELISA assay screening test that is 99% sensitive for *Schistosoma mansoni* and 90% sensitive for *S. hematobium* (*10*). We confirmed FAST-ELISA–positive samples using an *S. mansoni*–specific immunoblot to detect species-specific antibody. We tested all samples using an *S. hematobium*–specific immunoblot, which is 95% sensitive and 99% specific for each species (*13*). We defined a positive test result as positive results by both tests, an indeterminate result as positive by FAST-ELISA but negative by immunoblot, and a negative result as negative by both tests.

We defined study participant exposures from 2 weeks before enrollment until second sample collection by 4 activity categories: no water-contact activity, swimming/wading only, kayaking/rafting only, and swimming/wading plus kayaking/rafting. We defined schistosome antibody seroconversion in participants as either being first-test–negative and second-test–positive, or being first-test–negative and second-test–indeterminate. We compared characteristics between groups using the χ^2^ test for categorical data and the Mann-Whitney test for continuous variables (*14*). We expressed the risk for infection as the proportion of persons in each activity category who had evidence of schistosome antibody seroconversion and calculated the Mantel-Haenszel χ^2^ test for trend (*14*). We performed all analyses using SAS version 9.1 (SAS Institute, Cary, NC, USA). The CDC Institutional Review Board and the Uganda Virus Research Institute approved this study.

We enrolled 150 study participants; 2 subsequently withdrew. Thirty-five (24%) participants were not followed up because their first blood test was positive; all of these persons reported previous exposure to fresh water in schistosomiasis-endemic countries. Of the remaining 113 persons eligible for follow-up, 69 (61%) provided a second blood sample. Persons who provided only 1 blood sample were more likely to be younger (p = 0.005) and female (p = 0.03) (Table 1).

**Table 1:** Characteristics of 113 recreational users of the upper Nile River who participated in a study to determine risk for schistosome infection, were eligible for study follow-up, and provided 1 or 2 blood samples, Uganda, 2007*

Characteristic	Provided 1 blood sample, no. (%), n = 44	Provided 2 blood samples, no. (%), n = 69	p value†
Male sex	14 (32)	34 (50)	0.03
Main reason for being at the Nile			
Rafter	20 (45)	32 (46)	0.4
Spectator at the competition	12 (27)	18 (26)	
Kayak competitor	9 (21)	8 (12)	
Other	3 (7)	11 (16)	
Region of residence			
Africa	6 (14)	16 (23)	0.6
Americas	12 (27)	16 (23)	
Europe	15 (34)	17 (25)	
Australasia	7 (16)	12 (17)	
None given	4 (9)	8 (12)	

*Median ages (ranges) of study participants were as follows: 1 blood sample provided: 25 y (18–41 y); 2 blood samples provided, 29 y (16–67 y); p = 0.005. †p values estimated by using the Mann-Whitney test for age and a χ^2^ test for all other variables.

Of 69 persons followed up, 23% had fever, 13% cough, 10% skin rash, and 10% abdominal pain; 8% reported prickling skin. None reported physician-diagnosed acute schistosomiasis. Twelve (17%) of the 69 persons with 2 blood samples had evidence of seroconversion. No seroconversions were identified among the 9 persons who reported no water-contact activities. Serologic data suggested that infection occurred in 1 (13%) of 8 reporting swimming/wading only; 4 (15%) of 26, kayaking/rafting only; and 7 (27%) of 26, swimming/wading plus kayaking/rafting (Table 2).

**Table 2:** Proportion of recreational users of the upper Nile River who had schistosome infection, Uganda, 2007*

Activity	No. infected/total (%)
No water-contact activity	0/9
Swimming/wading only	1/8 (13)
Kayaking/rafting only	4/26 (15)
Kayaking/rafting and swimming/wading	7/26 (27)
All study participants	12/69 (17)

*Activity categories are mutually exclusive. χ^2^ test for trend p = 0.06

Of 106 persons for whom data were recorded, 19 (18%) reported self-medicating with praziquantel while at the kayaking competition. Of the 12 participants with evidence of seroconversion, 6 had data recorded about self-medication, none of whom took praziquantel.

## Conclusions

Approximately one fifth of persons with recreational exposure to water on the upper Nile River in Jinja District showed evidence of schistosome antibody seroconversion. Infection occurred among persons who reported swimming/wading only, kayaking/rafting only, and both activities, which refutes the belief that exposure to fast-moving water presents a low risk for schistosomiasis.

Exposure to schistosomes is likely to be highest in slow-moving water near riverbanks; thus, persons who go rafting may be at highest risk while putting their kayaks/rafts into and taking them out of the river. Although we were unable to estimate the risk for infection attributable to fast-moving white-water exposure alone, we did find that persons who reported swimming/wading and kayaking/rafting had the highest risk, possibly because of increased duration of exposure (*4*).

Eighteen percent of study participants reported self-medicating with praziquantel immediately or shortly after river water exposure. However, they would not have been protected against schistosomiasis because praziquantel acts against mature schistosome parasites and thus is most effective if taken after the parasite has developed to the adult stage, which is 4–6 weeks after infection. Local advice about using praziquantel to prevent schistosomiasis may not be appropriate; because indigenous populations have ongoing exposure, timing of treatment is not as critical. Travelers with discrete freshwater exposures in schistosomiasis-endemic countries should consult a travel medicine physician. In addition, information could be made available to pharmacies, rafting companies, and travelers about when to take praziquantel.

Our study had several limitations. The study cohort was a convenience sample, and participants might not have had equal chance of being enrolled. Use of this sample may have introduced bias, although whether any such bias would contribute to overestimation or underestimation of risk is unclear. Because schistosome antibody tests do not differentiate newly acquired infection, we excluded persons with first-test–positive results from the study follow-up. However, if these persons were more likely to have had a higher risk for infection, excluding them would have led us to underestimation risk for infection.

More than 12,000 persons take rafting trips in Uganda each year. Many travelers do not follow advice to avoid freshwater activities in schistosomiasis-endemic countries (*15*). Travelers should be made aware that white-water exposure presents a risk for schistosomiasis and that treatment with praziquantel should be at least 4–6 weeks after last exposure, preferably under the direction of a travel medicine physician.

## Supplementary Material

Appendix Table Persons and public health agencies assisting with the collection of follow-up blood samples in a study of schistosome infection among recreational users of the upper Nile River, Uganda, 2007*
