# Supplementary material for: Schistosomiasis among Recreational Users of Upper Nile River, Uganda, 2007
Source: Emerg Infect Dis. 2010 May;16(5):866–8. doi: 10.3201/eid1605.091740 (PMC2954006; doi:10.3201/eid1605.091740)
Supplement: Appendix Table — Persons and public health agencies assisting with the collection of follow-up blood samples in a study of schistosome infection among recreational users of the upper Nile River, Uganda, 2007* [file 09-1740_appT-s1.pdf]

**Appendix Table.** Persons and public health agencies assisting with the collection of follow-up blood samples in a study of schistosome infection among recreational users of the upper Nile River, Uganda, 2007*

| Name | Public health agency |
|---|---|
| United States | |
| Sue Jenkerson | Alaska Department of Health and Social Services |
| Jane Conard | Mat-Su Public Health Center |
| Kanta Sircar | Los Angeles County Department of Public Health |
| Amy Karon | California Department of Public Health |
| Duc Vugia | California Department of Public Health |
| Douglas Hatch | California Department of Public Health |
| Frank Alvarez | Santa Barbara County Public Health Department |
| Paige Batson | Santa Barbara County Public Health Department |
| Ken Gershman | Colorado Department of Public Health |
| Kate Lujan | Colorado Department of Public Health |
| Alice Guh | Connecticut Department of Health |
| Paul Melstrom | Georgia Department of Human Resources |
| Laurel Garrison | Georgia Department of Human Resources |
| Randy Nett | Idaho Department of Health and Welfare |
| Kathy Reynolds | Southeastern District Health Department |
| Alfreda DeMaria | Massachusetts Department of Public Health |
| Patricia Kludt | Massachusetts Department of Public Health |
| Kathleen Gilmore | Massachusetts Department of Public Health |
| Steven Helgersen | Montana Department of Public Health and Human Services |
| Ellen Leahy | Montana Department of Public Health and Human Services |
| Shelly Meyer | Montana Department of Public Health and Human Services |
| Vivian Mears | New Hanover County Health Department |
| Myra Brinson | Raleigh State Laboratory |
| Ning An | Oregon Department of Human Services |
| Emilio Debess | Oregon Department of Human Services |
| Susan Donnelly | Hood River Health Department |
| John Dunn | Tennessee Department of Health |
| John Su | Texas Department of State Health Services |
| Thi Dang | Texas Department of State Health Services |
| Canada | |
| Diane MacDonald | Public Health Agency of Canada |
| Denise Werker | Public Health Agency of Canada |
| Glennis Doiron | Calgary Laboratory Services |
| Muhammad Morshed | British Colombia Centre for Disease Control |
| Laura MacDougall | British Colombia Centre for Disease Control |
| James Aw | Medcan Clinic, Ontario |
| Beth Swift | Medcan Clinic, Ontario |
| Australia | |
| Anthony Moore | Health Protection Service, ACT Health |
| Riemke Kampen | Health Protection Service, ACT Health |
| Tiffany Savli | ACT Pathology, ACT Health |
| Jeremy McAnulty | Communicable Diseases Branch, New South Wales |
| Hanisah Corner | Center for Epidemiology and Research, New South Wales |
| Rogan Lee | Westmead Hospital, New South Wales |
| Christine Selvey | Communicable Diseases Branch, Queensland Health |
| Avner Misrachi | Department of Health and Human Services, Tasmania |
| David Coleman | Department of Health and Human Services, Tasmania |
| Margaret Hill | Department of Health and Human Services, Victoria |
| Megan Scully | Western Australia Department of Health |
| New Zealand | |
| Simon Baker | Auckland Regional Public Health Service |
| Don Bandaranayake | Population Health Directorate, Ministry of Health |
| Alistar Humphrey | Canterbury District Health Board |
| Ramon Pink | Canterbury District Health Board |
| Jill Geary | Canterbury District Health Board |
| Belinda Loring | Toi Te Ora Public Health Board |
| Anita Bell | Waikato District Health Board |
| Daphne Fairfood | Diagnostic Medlab Limited |
| Europe | |
| Tizza Zomer | Swedish Institute for Infectious Disease Control, Sweden |
| Esther Kissling | Institut Scientifique de Santé Publique, Belgium |
| Sabrina Bacci | Statens Serum Institut, Denmark |
| Kaisa Mäkinen | Lapland Central hospital, Finland |
| Laura Pakarinen | National Public Health Institute, Finland |
| Gudrun Bettge-Weller | Fachgebiet Infektiologische Diagnostik, Germany |

| | |
|---|---|
| Anja Hauri | Hesse State Health Office, Germany |
| Joan O'Donnell | HSE–Health Protection Surveillance Centre, Ireland |
| Tone Brunn | Norwegian Institute of Public Health, Norway |
| Christina Furtado | Instituto Nacional de Saúde Dr Ricardo Jorge, Portugal |
| Eugenio Cordeiro | Administração Regional de Saúde do Centro, Portugal |
| Kitty Smith | Health Protection Scotland, Scotland |
| Titia Kortbeek | Netherlands Laboratory for Infectious, Netherlands |
| Praveen Sebastianpillai | Health Protection Agency, England |
| Jane Jones | Health Protection Agency, England |
| Lorenzo Pezzoli | Health Protection Agency, England |
| Richard Pebody | European Programme for Intervention Epidemiology Training, European Centre for Disease Prevention and Control |
| Viviane Bremer | European Programme for Intervention Epidemiology Training, European Centre for Disease Prevention and Control |

*ACT, Australian Capital Territory; HSE, Health Service Executive.
